# Virtual Surgical Planning for Management of Acute Maxillofacial Trauma

**DOI:** 10.3390/cmtr18010018

**Published:** 2025-02-21

**Authors:** Kyle W. Singerman, Megan V. Morisada, J. David Kriet, John P. Flynn, Clinton D. Humphrey

**Affiliations:** Department of Otolaryngology—Head and Neck Surgery, University of Kansas Medical Center, 3901 Rainbow Boulevard, Kansas City, KS 66160, USA; ksingerman@kumc.edu (K.W.S.); megan.morisada@gmail.com (M.V.M.); dkriet@kumc.edu (J.D.K.); jflynn2@kumc.edu (J.P.F.)

**Keywords:** maxillofacial, trauma, mandibular, midface, ballistics, virtual surgical planning

## Abstract

Study design: A retrospective case series. Objective: The management of acute complex maxillofacial trauma is challenging. The intricate maxillofacial anatomy coupled with the significant functional and aesthetic repercussions of traumatic facial injuries necessitate meticulous preoperative preparation and operative precision to minimize patient morbidity. The severe displacement of bone fragments, abnormal occlusion, comminution, and the involvement of multiple skeletal subsites further complicate the restoration of premorbid function and appearance. While previously recognized as a valuable tool for managing oncologic defects, orthognathic surgery, and for the correction of secondary deformities following maxillofacial trauma, virtual surgical planning (VSP) has now emerged as a viable tool for treating select patients following acute complex maxillofacial trauma. Methods: A retrospective chart review of all the complex facial trauma patients treated using VSP services over a 21-month period. Results: Multiple VSP services were used in the primary repair of complex facial trauma, with occlusal splints, pre-contoured plates, and 3D printed models being utilized most frequently. Conclusions: Our experience with VSP for primary maxillofacial trauma repair has helped us to identify specific indications for the use of VSP in this setting.

## 1. Background

Virtual surgical planning (VSP) encompasses a broad set of tools used for the planning and simulation of the pre-, intra-, and postoperative phases of facial trauma repair. A surgeon—often with the assistance of an engineer working for a third-party vendor—uses software to virtually reduce fractures, establish occlusion, build 3D stereolithographic models, and create patient-specific plates, implants, dental occlusal splints, and cutting/drilling guides. The efficacy of VSP has been proven in craniomaxillofacial surgery, including in head and neck oncologic reconstruction [1,2,3,4], orthognathic surgery [5,6,7,8], and the correction of secondary maxillofacial trauma deformities [9,10,11,12,13,14,15].

Regarding the use of VSP in primary maxillofacial trauma surgery, the existing literature consists of case reports and small case series describing the use of VSP for individual facial subsites (e.g., ZMC, orbit) [16,17,18,19] and atrophic mandible fracture repair [20,21,22]. Other publications either discuss a singular VSP service [23,24,25,26] or provide a broad review of VSP in the context of maxillofacial trauma [27,28,29]. There is a paucity of literature proposing guidelines and specific indications for the use of VSP services for the primary repair of complex maxillofacial trauma [4]. Historically, one barrier to using VSP in the acute setting has been the extended time required to produce custom models and implants. Some vendors have needed up to 14 days for the fabrication of models or implants from whenever they acquire the computed tomography (CT) images for the patient [30,31]. This interval was considered excessive for the management of acute facial trauma, and some surgeons developed their own ‘in-house’ VSP protocols [20,21]. Unfortunately, not all surgeons have the requisite skills, and access to the equipment required for VSP may be limited primarily to academic centers with sufficient economic and technical resources. Fortunately, the logistical and technological improvements over recent years have shortened the time required to obtain commercial VSP services and implants. Our aim is to share the following about our experience with VSP:

1. Provide an overview of useful VSP services for patients with complex maxillofacial trauma.
2. Describe a case series of ten patients with severe maxillofacial trauma for which commercial VSP services were successfully implemented during their initial hospital admission.
3. Propose indications to consider VSP when treating acute maxillofacial trauma.

## 2. VSP Services for Maxillofacial Trauma

### 2.1. Virtual Fracture Reduction

Computer-aided 3D design software is used to visualize bone fragments, simulate reduction, and assess any bony defects. The surgeon can also evaluate and determine how occlusion will be re-established prior to surgery.

### 2.2. Occlusal Splints

An occlusal splint is a customized thin acrylic plate onto which the incisal and occlusal surfaces of a patient’s teeth can be firmly seated when placing a patient into a maxillomandibular fixation. The value of the splint depends on how precisely it fits the surfaces of an individual’s teeth. While less familiar to and less frequently used by some facial trauma surgeons without a dental background, occlusal splints are often used for establishing the desired occlusion during orthognathic surgery. High-quality occlusal splints can be an indispensable tool for restoring premorbid occlusion following acute facial trauma such as concomitant mandibular and maxillary fractures especially with a palatal split. Multiple disruptions of the dentition and a lack of stable dental arches in these scenarios make it extremely difficult for the surgeon to accurately determine premorbid occlusion. In the past, obtaining dental impressions manually using a substance such as alginate was always required to create occlusal splints. Obtaining impressions following acute facial trauma can be near impossible due to intubation, patient location, comorbid soft tissue injuries, loose dentition, and/or severe bony comminution. Some facial trauma surgeons, particularly those without a dental background, also lack the skills and/or resources to obtain impressions and make splints. VSP can make occlusal splints more accessible for use in acute facial trauma patients by using data from a patient’s fine cut (0.58 mm at our institution) CT maxillofacial images to print patient-specific occlusal splints from 3D renderings of occlusal surfaces and ideal post-reduction occlusion. Multiple case reports and small case series have begun to show that occlusion and facial symmetry can be restored using 3D-printed occlusal splints based on imaging in primary trauma reconstruction [24,25,32,33].

### 2.3. Patient Models

Stereolithographic patient-specific models can be 3D printed and sterilized to have on the operative field based on either ideal post-reduction modeling using fragment pieces or mirrored over the sagittal plane from the contralateral “normal” side in hemifacial trauma. In the absence of normal contralateral anatomy that can be used for mirroring, VSP can still be used for the virtual reduction of fractures, the assessment of symmetry, and the production of a useful model. These models can serve as a reference for a 3D comprehension of bone fragment orientation, an ideal facial contour, an anatomic reduction, and occlusion intraoperatively. The model can also provide a surface on which the surgeon can contour stock plates on the operative field prior to implantation [25,27,34].

### 2.4. Patient-Specific Plates and Other Anatomic Implants

The use of pre-contoured surgical plates is common during microvascular reconstruction of large mandibular defects due to oncologic resection or following ballistic trauma [31,35]. More recently, using pre-contoured plates has been proposed for treating isolated mandibular fractures [36], orbital fractures [37], and ZMC fractures [38]. At our institution, we have found patient-specific 3D printed titanium plates to be useful for the treatment of patients with comminuted mandibular, Le Fort, and palatal fractures. These 3D printed plates are more rigid and allow for the creation of nontraditional plate shapes and sizes when desired. Moreover, 3D printed plates can be created for the midface in novel configurations that cannot be replicated with stock plates.

In addition to custom plates, other patient-specific implants used to restore maxillofacial skeletal contour can be obtained in conjunction with VSP. Patient-specific anatomic implants are frequently used to restore the volume and contour to sites such as the orbit, malar region, and forehead [39,40,41]. These implants are available in a variety of materials (e.g., polyether-ether-ketone or PEEK) and are more frequently used to correct secondary traumatic deformities than for acute facial trauma in our practice.

## 3. Patients and Methods

A retrospective review was carried out over a 21-month period from January 2021 to October 2023. All patients with significantly comminuted facial fractures for which VSP services were utilized during the primary repair were included. Further detail surrounding the inclusion criteria and patient selection is described below in the “Indications to consider VSP when treating acute facial trauma” section. Facial trauma patients with non-comminuted fracture patterns and fractures limited to one subsite did not require VSP services and were accordingly excluded. This study was approved by the Institutional Review Board (University of Kansas Medical Center, Study00150801).

Commercially available VSP services were utilized in the primary repair of 10 complex maxillofacial trauma cases (Table 1). The workflow for these services consisted of a virtual meeting with a third-party vendor and engineer to virtually reduce the fractures prior to product acquisition. The patients had a median age of 38 (range 18–72) and all were males. The injuries were most commonly due to gunshot wounds (GSWs; *n* = 6), followed by motor vehicle collisions (MVCs; *n* = 2), a ground level fall (*n* = 1), and an occupational injury where a patient was hit in the face with a forklift (*n* = 1). Occlusal splints were the most utilized VSP service (*n* = 7) followed by 3D printed surgical plates (*n* = 4) and patient-specific 3D models (*n* = 4). Multiple injury subsites were common, with 8 of 10 patients having multiple concomitant injury patterns and involved anatomic subsites (Table 2). There were eight mandibular fractures (80%), six palatal fractures (60%), four orbital fractures (40%), four Le Fort I fractures (40%), three naso-orbito-ethmoid (NOE) fractures (30%), and two patients had combined Le Fort II, Le Fort III, and ZMC fractures. One patient had a frontal sinus fracture. Surgical intervention using VSP-derived services required an average of 8.6 days (range 5–13) following an initial consultation. Operative times lasted an average of 276 min (range 130–450) (Table 2). There was one instance of an initial operative fracture repair attempt that needed to be deferred until a VSP—derived occlusal splint and 3D printed plates could be obtained due to the inability to appropriately reduce a mandibular fracture secondary to posterior symphyseal cortex and condylar splay.

Postoperative complications occurred in 7 of 10 patients, including maxillary osteonecrosis (*n* = 2), loose hardware (*n* = 2), orocutaneous fistula (*n* = 2), and a nasal obstruction due to synechiae and bone fragments (*n* = 1) (Table 2). Five of these seven complications required a return to the operating room (OR), including two instances of microvascular reconstruction for maxillary osteonecrosis. The patients had a median follow-up duration (i.e., the interval between the initial surgery and the most recent facial trauma clinic visit) of 370 days (range 33–1171 days).

## 4. Selected Illustrative Cases

### 4.1. Case 1 (Occlusal Splint)

**Figure 1:**
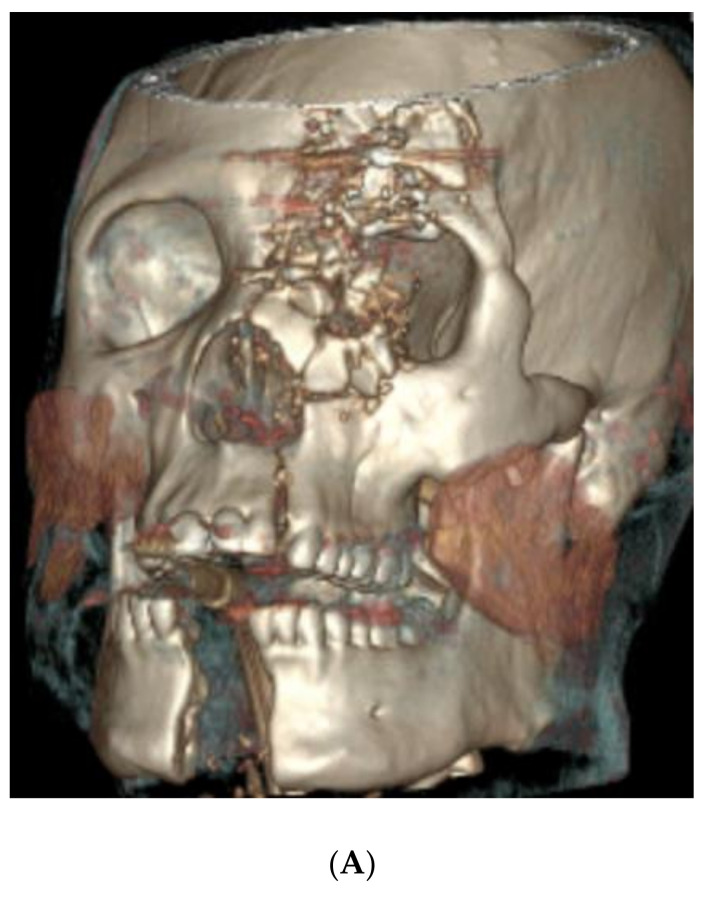

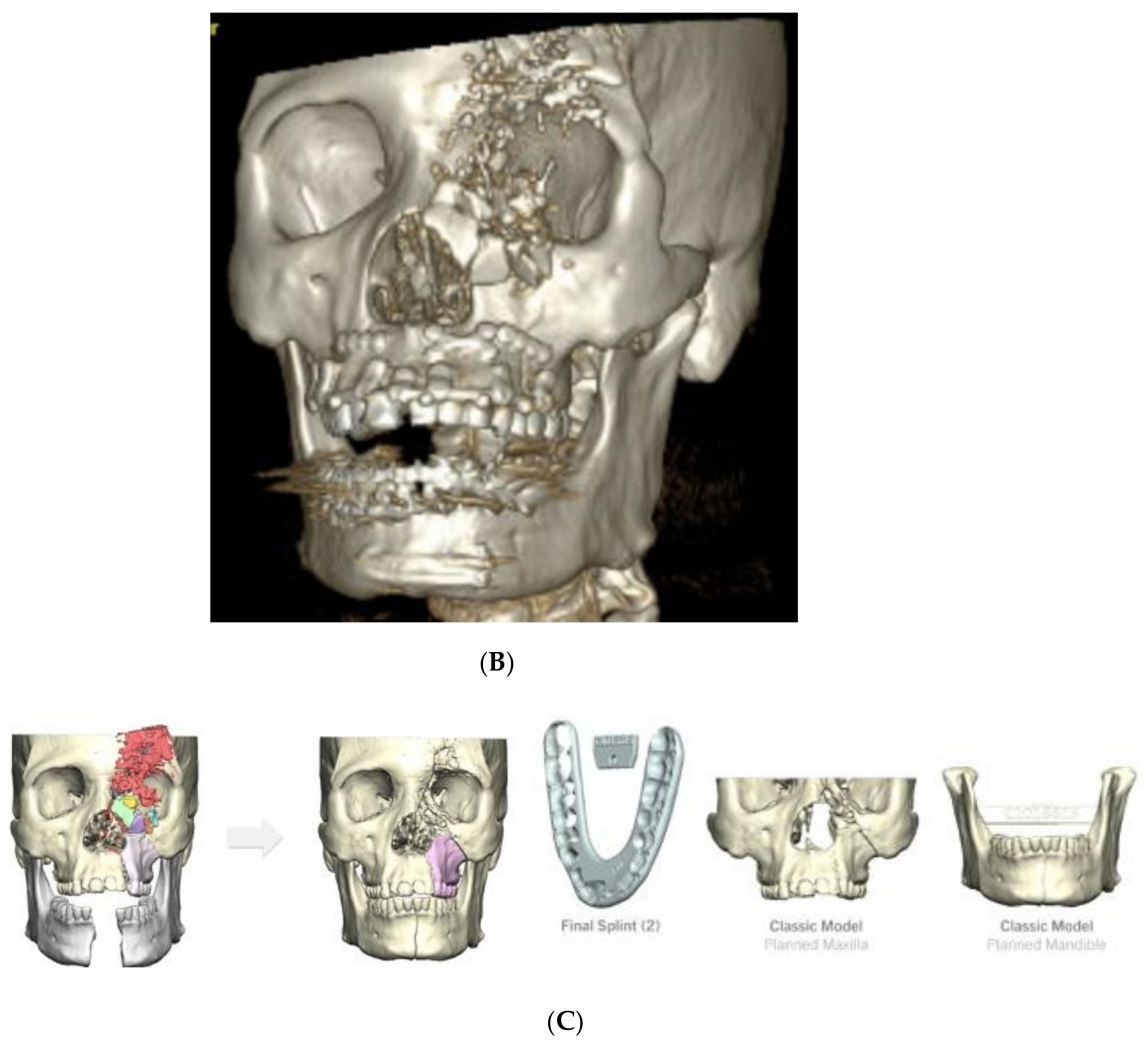
(**A**) Preoperative 3D CT demonstrating (but not limited to) panfacial fractures including Le Fort levels I/II maxillary fractures, transverse palatal fracture, and left mandibular symphyseal fracture; (**B**) postoperative 3D CT demonstrating fracture reduction and internal fixation. (**C**) Virtual fracture reduction with demonstration of occlusal splint and 3D printed models of ideal post-reduction maxilla and mandible.

A 34-year-old male presented after a self-inflicted GSW with a trajectory from the right chin to the left orbit (Figure 1A,B). Bony trauma included the following:

- An extensive calvarial fracture involving the left frontal sinus and left cribriform plate;
- Comminuted fractures of the left medial, superior, and inferior orbital walls;
- Left Le Fort I/II maxillary fractures;
- A comminuted transverse palatal fracture;
- A left mandibular symphyseal fracture (with 2.4 cm diastasis between the fracture segments);
- A comminuted nasal bone fracture.

Operative intervention took place seven days after the initial consultation. The surgery consisted of an open reduction and internal fixation (ORIF) of the mandibular symphyseal and maxillary fractures, intermaxillary fixation, ORIF of the left orbital floor, and medial wall fractures, and an orbitotomy with foreign body removal. The mandibular fracture was exposed through a transoral vestibular incision. A VSP-derived occlusal splint with intermaxillary fixation was used to confirm adequate reduction (Figure 1C). Mandible symphyseal fixation was carried out using a lag screw technique. A transoral vestibular incision was used to expose the three maxillary fracture lines and these fractures were repaired using double-Y and four-hole plates while the patient was maintained in maximal intercuspation occlusion. Arch and hybrid bars were placed along the maxillary and mandibular dentition for postoperative stabilization. Because of the unstable concomitant maxillary and mandibular fractures with multiple mobile segments, the use of a VSP-derived occlusal splint made it easier to find and maintain premorbid occlusion during fracture fixation.

### 4.2. Case 2 (Occlusal Splint, 3D Model)

**Figure 2:**
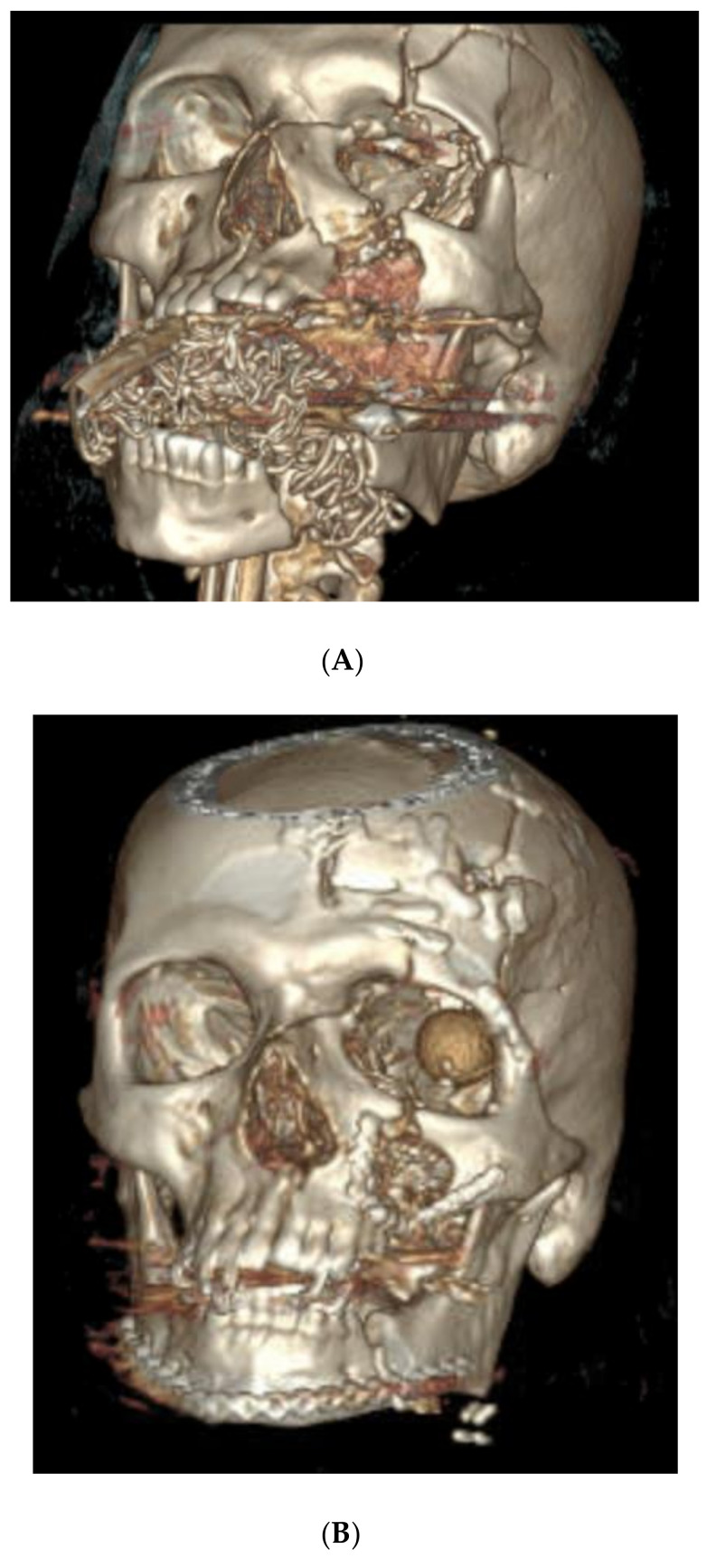

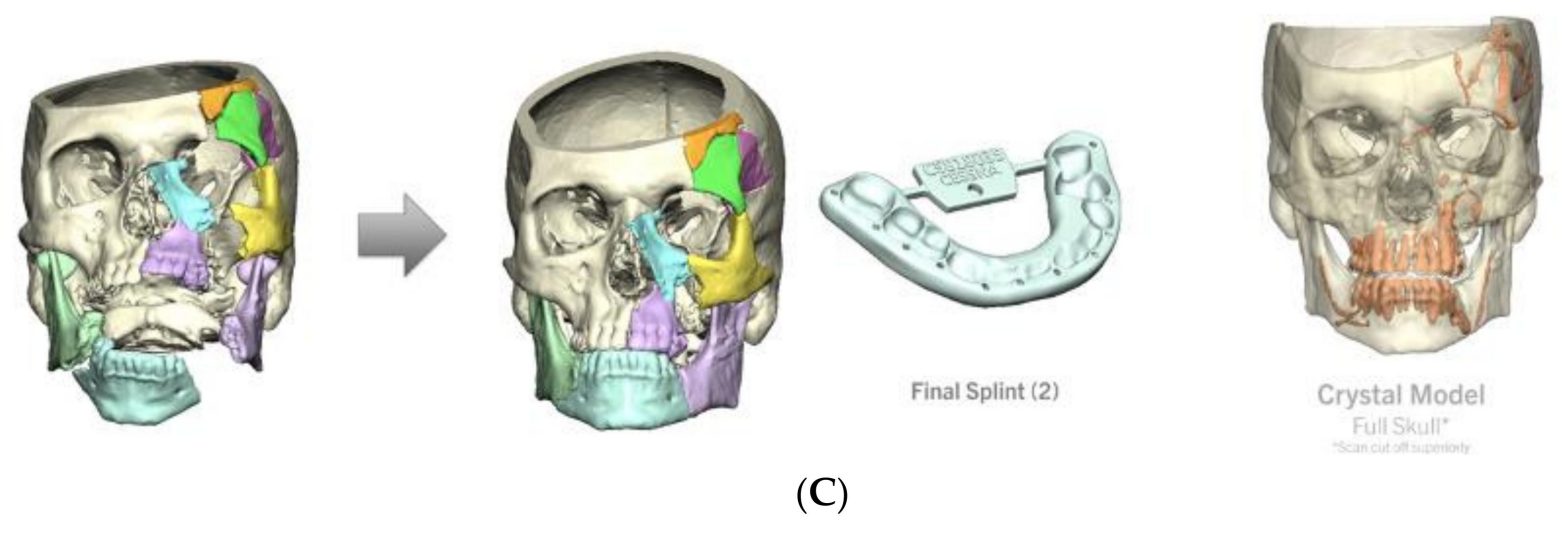
(**A**) Preoperative 3D CT demonstrating (but not limited to) left Le Fort levels I/II/III maxillary fractures, comminuted left maxillary fracture involving the hard palate with mobile palate segment, and bilateral comminuted and displaced mandibular body fractures; (**B**) postoperative 3D CT demonstrating fracture reduction and internal fixation. (**C**) Virtual fracture reduction with demonstration of occlusal splint and 3D printed model of ideal post-reduction maxilla and mandible.

A 72-year-old male presented with a self-inflicted GSW to the submental region. Bony trauma included the following (Figure 2A,B):

- Bilateral calvarial and skull base fractures;
- Comminuted left orbital fractures;
- Left Le Fort I/II/III maxillary fractures;
- A comminuted left maxillary fracture involving the left and right hard palate with a mobile palate segment;
- Bilateral comminuted, displaced, open mandibular body fractures;
- Bilateral comminuted nasal fractures.

The initial surgery consisted of a tracheotomy and the selective repair of soft tissue lacerations. The next operative intervention proceeded eight days following the initial consultation and included ORIF of the left maxillary fractures and mandibular fractures, and an enucleation by ophthalmology. A transoral approach was attempted for mandibular fracture repair; however, due to the immobility of the fracture segments and tongue edema, a transcervical approach was executed. An apron incision in the neck was used to expose three large segments of the significantly displaced mandibular bone. The VSP-derived occlusal splint was then utilized to establish premorbid occlusion, and the patient was placed in intermaxillary fixation. The bilateral mandibular fractures were reduced, and a VSP-derived crystal model was used to bend the stock mandibular reconstruction plate intraoperatively, which was placed along the inferior border of the mandible (Figure 2C). A bicoronal incision was used to access the Le Fort III fractures, during which time brain tissue was encountered and neurosurgery was consulted for frontal calvarial reconstruction intraoperatively. The zygomaticofrontal suture line was reduced and fixated, as were the lateral and medial buttresses of the maxilla. The soft tissue defects were closed to the maximal extent, and the VSP-derived occlusal splint was left in place postoperatively with rigid maxillomandibular fixation to help stabilize the maxillary and palatal fractures. Roughly two weeks later the occlusal splint was removed, and the patient was placed in elastics. At this time, his occlusion was found to be stable and reproducible. Given the severe displacement of the fractures as well as the involvement of both dental arches, the use of VSP to preoperatively plan ideal reductions and mirror the contralateral normal side was critical. This allowed for the creation of a crystal model to allow the intraoperative pre-contouring of plates as well as the implementation of an occlusal splint to ensure accurate premorbid occlusion.

### 4.3. Case 3 (Patient 3D Model, Occlusal Splint, 3D Printed Plate)

**Figure 3:**
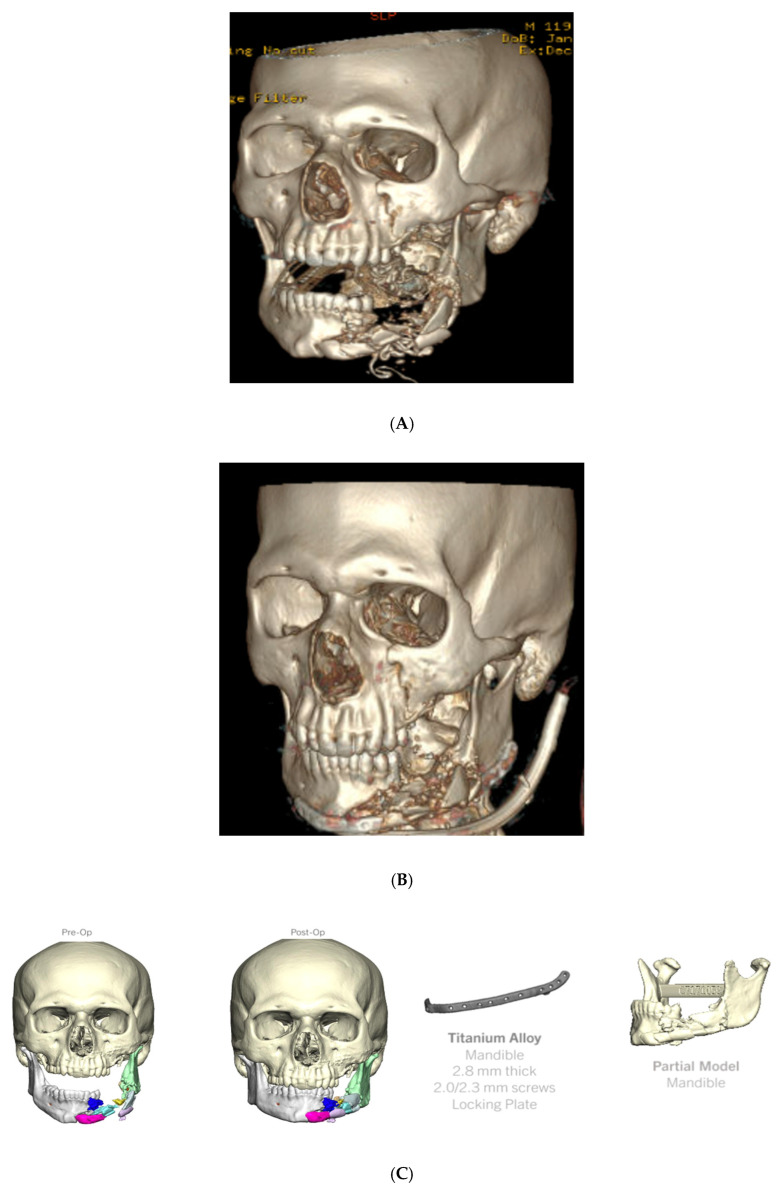
(**A**) Preoperative 3D CT demonstrating (but not limited to) comminuted maxillary fractures involving hard palate, and an extensively comminuted fracture of the left mandibular body extending to the parasymphysis; (**B**) postoperative 3D CT demonstrating fracture reduction and internal fixation. (**C**) Virtual fracture reduction with demonstration of patient-specific 3D printed mandibular plate and 3D printed model of ideal post-reduction mandible.

A 23-year-old male presented between Christmas and New Year’s with a self-inflicted GSW to the left face with resultant bony trauma including the following (Figure 3A,B):

- Left orbital floor and lateral orbital rim fractures;
- Comminuted maxillary fractures with involvement of the hard palate and retained bullet fragments in the maxillary sinus;
- An extensively comminuted open fracture of the left mandibular body extending to the parasymphysis;
- Extensive soft tissue and dental injuries with retained ballistic fragments.

Operative intervention proceeded 13 days after the initial consultation and included mandibular ORIF with a patient-specific 3D printed 2.8mm reconstruction plate (Figure 3C). The fractures were exposed using a transcervical-modified Apron incision in addition to a large intraoral defect. The patient was placed into occlusion using an occlusal splint and dental occlusion ties. The fractures were reduced with guidance from the 3D printed reconstruction plate, which was secured in place with bicortical locking screws with the screw length predetermined using VSP. Stable and reproducible occlusion was confirmed, and the occlusal splint was removed at the end of the case. Due to the significant comminution of the mandibular fracture, there were no solid bony fragments to adequately guide the reduction. For this reason, the utilization of a VSP-derived 3D printed plate in addition to a VSP-derived occlusal splint allowed for greater intraoperative precision. This patient was taken back to the operating room roughly 1.5 years later to remove loose screws and contour a prominent bone fragment that was lateral to the plate along the left mandibular body. The entire reconstructive plate and screws were removed at this time with complete bone healing observed intraoperatively.

## 5. Indications to Consider VSP When Treating Acute Facial Trauma

The authors propose the following indications for the use of VSP for primary reconstruction of maxillofacial trauma:

1. VSP can be used to virtually assess and restore occlusion when comminuted panfacial injuries involve both the midface and mandible, especially when a symphyseal mandible fracture is present that can lead to unintentional facial widening during fracture reduction. Three-dimensional models will assist with plate bending and fracture reduction. Occlusal splints can help the surgeon achieve stable maxillomandibular fixation in these patients intraoperatively to guide fracture reduction.
2. Palatal split fractures make restoration of stable occlusion extremely difficult. VSP-derived occlusal splints can be used both intraoperatively and, if needed, postoperatively to help reduce and/or stabilize these fractures.
3. When ballistic trauma results in excessive comminution and pulverization of bone, VSP can be used to assist with fracture reduction and, if needed, for the production of custom implants that span the areas of bone loss.
4. Limited or poor dentition makes traditional maxillomandibular fixation techniques ineffective as a guide to fracture reduction. The virtual assessment of both occlusion and fracture reduction using VSP is valuable in this setting. An occlusal split can also make stable maxillomandibular fixation possible in many cases.

Our proposed indications build on the prior work from Chen et al. who suggested that 3D-printed occlusal splints may be particularly useful in patients with more than three bone fragments on the mandible or maxilla or more than four bone fragments involving the entire maxillomandibular complex [32].

## 6. Discussion

The restoration of facial contour and occlusion is challenging following complex maxillofacial trauma. VSP can potentially improve operative efficiency and patient outcomes for difficult cases. While all VSP services are valuable, it is the authors’ experience that occlusal splints and 3D models can be most rapidly obtained and are more frequently used VSP tools for acute injuries. It remains a priority for us to perform repair during the patient’s initial admission whenever possible; both splints and 3D models require less time to fabricate and are of a significantly lower cost than custom implants such as 3D-printed titanium plates. When feasible, we do prefer custom-fabricated titanium plates in many situations due to their superior rigidity and contour; however, contouring stock plates to a 3D model can also be effective.

Our case series focused on complex maxillofacial trauma in the acute setting, where VSP has served as a significant boon in the repair of otherwise very difficult fracture patterns. While VSP could certainly be useful in less complex cases, it is less likely to improve the outcomes for these injuries, and the cost must also be a consideration. Two prior studies have investigated the cost effectiveness of VSP in facial trauma repair and found a lower overall cost; however, they were both utilizing “in-house” protocols rather than the commercial vendors used in our case series [42,43].

The average procedure time for this case series was 276 min. While the heterogeneity of the injuries in our case series makes it difficult to extrapolate any definitive conclusions regarding reduction in operative time, it is likely that in complex facial trauma cases, VSP services reduce operative time, leading to decreased costs. We perceive that the following VSP benefits save time intraoperatively:

1. Following a virtual planning session, surgeons may have a better understanding of how bone fragments will be reduced prior to surgery.
2. Custom occlusal splints and plates may guide bony reduction insofar as the surgeon will not be relying solely on the alignment of partially visible fracture lines and/or sometimes unstable occlusion during the procedure.
3. Bending plates to a 3D model produced with VSP is easier than trying to bend plates to the patient via the limited access provided by surgical approaches.

In our series, primary repair occurred at an average of 8.6 days from an initial consultation, a relatively similar figure to an average of 6.5 days from presentation-to-repair that has previously been observed in a large cohort of facial trauma patients, with the fracture severity as the greatest predictor of delays in operative intervention [44]. In case 3, a 13-day delay from the initial consultation to the primary repair may have been a result of lag time in obtaining VSP services due to gaps between “business” days over the holidays, which continue to be a consideration when electing to use VSP. With respect to complications, this was a highly complex patient population, many of whom suffered from facial GSWs. Furthermore, follow-ups were both heterogeneous and limited. The 50% rate of complications that required a revision surgery is inherently higher than it would be for a more “standard” subset of facial trauma patients. No occlusal complications were described, and the two instances of maxillary osteonecrosis that occurred in the complex GSW patients were likely independent of the decision to utilize VSP services.

This case series intends to introduce VSP as a tool that the authors believe can be useful in the repair of acute complex maxillofacial trauma. The limitations include a relatively small sample size and primarily empirical data regarding VSP use in this setting. If VSP becomes more widely adopted, future studies could certainly seek to compare operative times or other outcomes between traditional and VSP-driven (both in-house and commercially available) repairs. While some in-house VSP processes might continue to exist, commercial VSP services are likely to be the more realistic option for most surgeons as availability, technology, and turnaround times continue to improve.

## 7. Conclusions

Primary reconstruction of severe maxillofacial trauma, especially combined and comminuted injuries of the maxillomandibular complex, in the acute setting is a complicated endeavor. Without significantly delaying surgical treatment, VSP services have the potential to help facial trauma surgeons repair the most complicated fractures.

## Figures and Tables

**Table 1:** Patient demographics.

Age at Surgery * (Years).	38 (range 18–72)
**Male Gender (*n*)**	10
**Mechanism (*n*)**	
GSW	6
MVC	2
GLF	1
Occupational	1
**VSP Used (*n*)**	
Occlusal splint	7
3D printed surgical plate	4
Patient 3D model	4
**Injuries (*n*)**	
Mandibular fracture	8
Palatal fracture	6
LeFort I	4
LeFort II	2
LeFort III	2
Orbital fracture	4
NOE	3
ZMC fracture	2
Frontal sinus fracture	2
Consult to OR Time * (days)	8.6 (range 5–13)
Length of Surgery * (minutes)	276 (range 130–450)
**Complication Rate**	70%
**Length of Follow-up ^&^ (days)**	370 (range 33–1171)

* Mean; ^&^ Median.

**Table 2:** Injuries, surgical details, and clinical course of individual patients.

Age	Etiology	Facial Fractures	Other Injuries	VSP Services	Consult to OR Time (Days)	Length of Surgery (Minutes)	Postoperative Complications and Interventions	Length of Follow-Up (Days)
27	GSW	Mandibular fracture (complex) LeFort II LeFort III Left orbital fracture (two-wall) Bilateral NOE	None	Pre-contoured plate (left mandibular ramus)	9	450	Oroantral fistula Necrotic maxillary and mandibular bone Midface reconstruction with fibula free flap	1171
69	Occupational	LeFort I Maxillary fracture (palate split)	Globe rupture	Occlusal splint Pre-contoured plate (maxillary spine)	8	281	Nasal obstruction due to synechiae and bone fragment To be addressed in OR	296
34	GSW	Mandibular fracture Maxillary fracture (complex) LeFort I Frontal sinus fracture	Orbital foreign body Eyelid lacerations	Occlusal splint 3D model (mandible and maxilla)	7	209	None	33
33	GSW	Mandibular fracture (complex) Maxillary fracture (complex, palate split) Left orbital fracture (two-wall) Left NOE Left ZMC	Eyelid lacerations	Occlusal splint	7	299	Osteonecrosis of maxilla Attempted fibula free flap, failed intraoperatively due to carotid injury; treated with obturator	500
38	MVC	LeFort I LeFort II LeFort III Bilateral orbital fractures (three-wall) Left NOE	Severe intracranial injury Left retrobulbar hematoma Rib fractures and pulmonary contusions Right hip fracture Multiple extremity fractures	Pre-contoured plates (zygomatico-frontal, infraorbital rim, LeFort I)	11	349	None	400
31	MVC	Mandibular fracture (complex)	Rib fractures and pulmonary contusions Femur fracture Spinal process fracture	Occlusal splint 3D model (mandible)	9	209	Orocutaneous fistula (intraoral to submental) Necrotic exposed mandible Bone and soft tissue debrided in OR	417
72	GSW	Mandibular fracture (complex) Maxillary fracture (complex, palate split) Left orbital fracture Left ZMC	Severe intracranial injury Left globe injury	Occlusal splint	9	433	None	48
23	GSW	Mandibular fracture (complex) Maxillary fracture (complex, palate split)	None	Occlusal splint Pre-contoured plate (mandible) 3D model (mandible)	13	130	Palpable bony overgrowth Loose screws All hardware removed in OR, prominent areas of bone drilled down; postoperative wound infection, treated medically	571
41	GSW	Mandibular fracture (complex)	None	3D model (mandible)	8	221	Orocutaneous fistula Treated medically	341
18	GLF	Mandibular fracture (simple) Maxillary fracture (palatal split) LeFort I	None	Occlusal splint	5	175	Loose plate (palate) Removed in OR	110

## Data Availability

Data is unavailable due to patient privacy.
